# Bis[(di­methyl­phosphor­yl)methan­amin­ium] tetra­chlorido­palladate(II)

**DOI:** 10.1107/S1600536813028067

**Published:** 2013-10-19

**Authors:** Guido J. Reiss

**Affiliations:** a Institut für Anorganische Chemie und Strukturchemie, Lehrstuhl II: Material- und Strukturforschung, Heinrich-Heine-Universität Düsseldorf, Universitätsstrasse 1, D-40225 Düsseldorf, Germany

## Abstract

In the crystal structure of the title compound, (C_3_H_11_NOP)_2_[PdCl_4_], (di­methyl­phosphor­yl)methanaminium (dpmaH^+^) cations are connected head-to-tail by strong N—H⋯O hydrogen bonds, forming inversion-related cyclic dimers. The square-planar [PdCl_4_]^2−^ counter-dianion is located about a center of inversion. The dications and the [PdCl_4_]^2−^ dianions are connected by medium–strong N—H⋯Cl hydrogen bonds, forming zigzag chains parallel to [001]. Somewhat weaker N—H⋯Cl hydrogen bonds connect the chains into a three-dimensional network.

## Related literature

For transition metal complexes built by the neutral dpma ligand, see: Kochel (2009 ▶). For simple dpmaH^+^ salts, see: Reiss & Jörgens (2012 ▶); Buhl *et al.* (2013 ▶); Lambertz *et al.* (2013 ▶); Reiss (2013*a* ▶). For dpmaH^+^ metal complexes, see: Reiss (2013*b* ▶,*c* ▶,*d* ▶). For some structures and applications of tetra­chlorido­palladate(II) salts, see: Willett & Willett (1977 ▶); Hardacre *et al.* (2001 ▶); Lee *et al.* (2004 ▶); Song *et al.* (2012 ▶); Vranec *et al.* (2012 ▶); Serpell *et al.* (2013 ▶). For graph-set analysis, see: Grell *et al.* (2002 ▶).
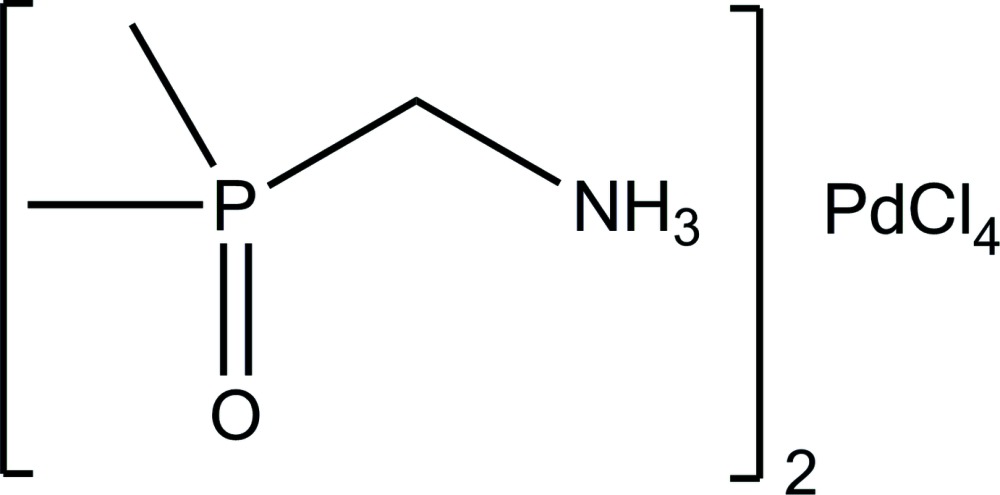


## Experimental

### 

#### Crystal data

(C_3_H_11_NOP)_2_[PdCl_4_]
*M* *_r_* = 464.39
Monoclinic, 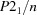

*a* = 9.3600 (3) Å
*b* = 7.81198 (19) Å
*c* = 11.9892 (3) Å
β = 110.110 (3)°
*V* = 823.21 (4) Å^3^
*Z* = 2
Mo *K*α radiation
μ = 1.96 mm^−1^
*T* = 290 K
0.18 × 0.12 × 0.11 mm

#### Data collection

Oxford Diffraction Xcalibur CCD diffractometer
Absorption correction: analytical [using a multifaceted crystal model (Clark & Reid, 1995 ▶)] *T*_min_ = 0.764, *T*_max_ = 0.850
26379 measured reflections
3595 independent reflections
3077 reflections with *I* > 2σ(*I*)
*R*_int_ = 0.035

#### Refinement

*R*[*F* ^2^ > 2σ(*F* ^2^)] = 0.022
*wR*(*F* ^2^) = 0.051
*S* = 1.09
3595 reflections
94 parameters
H atoms treated by a mixture of independent and constrained refinement
Δρ_max_ = 0.46 e Å^−3^
Δρ_min_ = −0.57 e Å^−3^

### 

Data collection: *CrysAlis PRO* (Oxford Diffraction, 2009 ▶); cell refinement: *CrysAlis PRO*); data reduction: *CrysAlis PRO*); program(s) used to solve structure: *SHELXS2013* (Sheldrick, 2008 ▶); program(s) used to refine structure: *SHELXL2013* (Sheldrick, 2008 ▶); molecular graphics: *DIAMOND* (Brandenburg, 2012 ▶); software used to prepare material for publication: *publCIF* (Westrip, 2010 ▶).

## Supplementary Material

Crystal structure: contains datablock(s) I, New_Global_Publ_Block. DOI: 10.1107/S1600536813028067/wm2775sup1.cif

Structure factors: contains datablock(s) I. DOI: 10.1107/S1600536813028067/wm2775Isup2.hkl

Additional supplementary materials:  crystallographic information; 3D view; checkCIF report

## Figures and Tables

**Table 1:** Hydrogen-bond geometry (Å, °)

*D*—H⋯*A*	*D*—H	H⋯*A*	*D*⋯*A*	*D*—H⋯*A*
N1—H12⋯Cl1	0.88 (2)	2.40 (2)	3.2220 (15)	155 (2)
N1—H11⋯O1^i^	0.86 (2)	1.89 (2)	2.7425 (17)	172 (2)
N1—H13⋯Cl1^ii^	0.84 (2)	2.73 (2)	3.3752 (15)	135.1 (18)
N1—H13⋯Cl2^ii^	0.84 (2)	2.82 (2)	3.5241 (16)	143.4 (18)

Symmetry codes: (i) 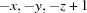; (ii) 
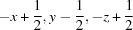
.

**Fig. 1.**
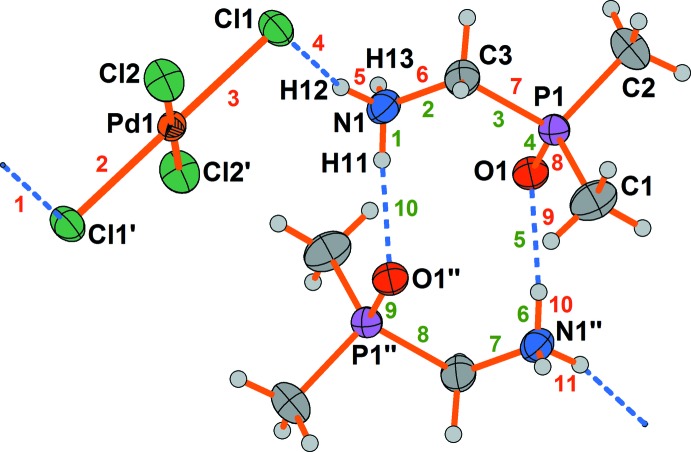
The cyclic dimer, built up from two dpmaH+ cations, is hydrogen-bonded to neighbouring [PdCl4]2- anions. Ellipsoids are drawn at the 50% probability level. The graph-set descriptors R22(10) and C33(11) are indicated by green and red numbers. [Symmetry codes: (') -x, -y, -z; ('') -x, -y, 1-z.]

**Fig. 2.**
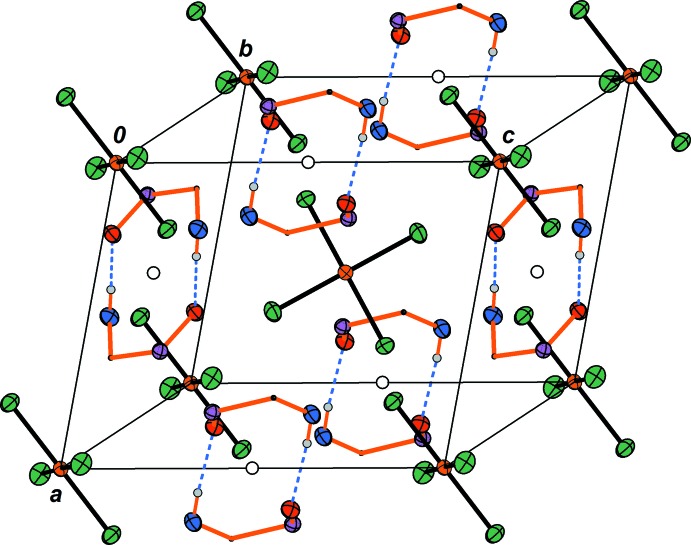
In the packing of the title structure the cations and anions occupy different centers of inversion. Therefore, the arrangement of cations and anions, respectively, represent body-centered sublattices. Ellipsoids are drawn at the 40% probability level; the methyl groups are omitted and the methylene group is shown in a wireframe style for clarity.

## supplementary crystallographic information

### 1. Comment

The synthesis of (dimethylphosphoryl)methanamine (dmpa) and its use as a bidentate *N,O*-ligand has been proved (Kochel, 2009 and literature therein). Furthermore, two transition metal complexes are reported in which the dpmaH^+^ cation acts as a monodentate ligand (Reiss, 2013**b*,c*). Recently, a series of dmpaH^+^ salts have been synthesized and structurally characterized. In all cases hydrogen bonds strongly affect the set-up of the crystal structures. In many cases the head-to-tail connection of two and more dpmaH^+^ tectons leads to hydrogen-bonded polymeric structures with the counter anions only very weakly attached (Buhl *et al.*, 2013; Lambertz *et al.* 2013; Reiss, 2013*a*), but there are structures which show the ability of the dpmaH^+^ tecton to form medium-strong hydrogen bonds with the surrounding cations and anions as well (Reiss & Jörgens, 2012; Reiss, 2013*d*).

For many decades, alkylaminium tetrachloridopalladate salts are of general interest (*e.g.* Willett & Willett, 1977). Imidazolium tetrachloridopalladate(II) salts have recently attracted much attention as potent precursors for preparation of catalytic metal nanoparticles (Serpell *et al.*, 2013), as solids that show thermochromism (Hardacre *et al.*, 2001) and may form liquid crystalline phases (Lee *et al.*, 2004). Furthermore, it should be mentioned that corresponding tetrachloridopalladate(II) salts have recently been used as pre-catalyst for the Suzuki reaction (Song *et al.* 2012).

The asymmetric unit of the structure of the title compound, (C_3_H_11_NOP)_2_[PdCl_4_], consists of one dpmaH^+^ cation and one half [PdCl_4_]^2-^ anion with the Pd(II) atom located on an inversion center (Wyckoff site: 2*a*). The bond lengths and angles of both ions are all in the expected ranges. Pairs of dpmaH^+^ cations are connected head-to-tail around centers of inversion (Wyckoff site: 2 *d*) forming cyclic dimers (Fig. 1). The cyclic dimers and the centrosymmetric [PdCl_4_]^2-^ anions are connected by medium-strong and charge-supported N—H···Cl hydrogen bonds (H···Cl = 2.41 (2) Å; Table 1), forming chains along [001]. The hydrogen bonding connection of the dicationic cycle can be classified by the first level graph-set descriptor *R*^2^_2_(10) (Grell *et al.* 2002), whereas the connection along the chain is represented by the second level graph-set descriptor *C*^3^_3_(11) (Fig. 1). Taking into account also weak bifurcated N—H···Cl hydrogen bonds (H···Cl: 2.73 (2) and 2.83 (2) Å), a three-dimensional network is obtained.

According to the occupation of two different centers of inversion by cyclic dimers and [PdCl_4_]^2-^ anions, respectively, the arrangement of each of them can be described as a body-centered sublattice (Fig. 2). Secondary Pd···Cl interactions perpendicular to the plane of the [PdCl_4_]^2-^ anion, known for related salt structures (*e.g.* Willett & Willett, 1977; Vranec *et al.*, 2012) are ruled out by the afore discussed packing scheme. In the title structure two methyl groups of two different (dpmaH)_2_^2+^ dications roughly occupy and block these axial positions at the Pd(II) atom (Fig. 1).

### 2. Experimental

In a typical experiment 0.25 g PdCl_2_ (1.4 mmol) were dissolved in 4 ml concentrated hydrochloric acid (37%) and 0.3 g (2.8) dpma was added to this yellow solution. Within a few days yellow crystals grow at the bottom of the vessel.

### 3. Refinement

All hydrogen atoms were identified in difference Fourier syntheses. The methyl groups were idealized and refined using rigid groups allowed to rotate about the P–C bond (AFIX 137 option of the *SHELXL-2013* program (Sheldrick, 2008)) with the *U*_iso_(H) = 1.5*U_eq_*(C). The hydrogen atoms at the CH_2_-group were idealized and treated as riding with *U*_iso_(H) = 1.2*U_eq_*(C). The coordinates of the hydrogen atoms at the NH_3_-group were refined freely simultaneously with individual *U*_iso_ values.

### Crystal data

**Table:**

(C_3_H_11_NOP)_2_[PdCl_4_]	*F*(000) = 464
*M**_r_* = 464.39	*D*_x_ = 1.874 Mg m^−^^3^
Monoclinic, *P*2_1_/*n*	Mo *K*α radiation, λ = 0.71073 Å
*a* = 9.3600 (3) Å	Cell parameters from 12224 reflections
*b* = 7.81198 (19) Å	θ = 3.2–35.4°
*c* = 11.9892 (3) Å	µ = 1.96 mm^−^^1^
β = 110.110 (3)°	*T* = 290 K
*V* = 823.21 (4) Å^3^	Block, yellow
*Z* = 2	0.18 × 0.12 × 0.11 mm

### Data collection

**Table:**

Oxford Diffraction Xcalibur CCD diffractometer	3595 independent reflections
Radiation source: (Mo) X-ray Source	3077 reflections with *I* > 2σ(*I*)
Graphite monochromator	*R*_int_ = 0.035
Detector resolution: 16.2711 pixels mm^-1^	θ_max_ = 35.0°, θ_min_ = 3.2°
ω scans	*h* = −15→14
Absorption correction: analytical [using a multifaceted crystal model (Clark & Reid, 1995)]	*k* = −12→12
*T*_min_ = 0.764, *T*_max_ = 0.850	*l* = −19→19
26379 measured reflections	

### Refinement

**Table:**

Refinement on *F*^2^	Secondary atom site location: difference Fourier map
Least-squares matrix: full	Hydrogen site location: difference Fourier map
*R*[*F*^2^ > 2σ(*F*^2^)] = 0.022	H atoms treated by a mixture of independent and constrained refinement
*wR*(*F*^2^) = 0.051	*w* = 1/[σ^2^(*F*_o_^2^) + (0.0178*P*)^2^ + 0.217*P*] where *P* = (*F*_o_^2^ + 2*F*_c_^2^)/3
*S* = 1.09	(Δ/σ)_max_ = 0.007
3595 reflections	Δρ_max_ = 0.46 e Å^−^^3^
94 parameters	Δρ_min_ = −0.57 e Å^−^^3^
0 restraints	Extinction correction: *SHELXL2013* (Sheldrick, 2008), Fc^*^=kFc[1+0.001xFc^2^λ^3^/sin(2θ)]^-1/4^
Primary atom site location: structure-invariant direct methods	Extinction coefficient: 0.0040 (4)

### Special details

**Table:**

Geometry. All e.s.d.'s (except the e.s.d. in the dihedral angle between two l.s. planes) are estimated using the full covariance matrix. The cell e.s.d.'s are taken into account individually in the estimation of e.s.d.'s in distances, angles and torsion angles; correlations between e.s.d.'s in cell parameters are only used when they are defined by crystal symmetry. An approximate (isotropic) treatment of cell e.s.d.'s is used for estimating e.s.d.'s involving l.s. planes.

### Fractional atomic coordinates and isotropic or equivalent isotropic displacement parameters (Å^2^)

**Table:**

	*x*	*y*	*z*	*U*_iso_*/*U*_eq_	
Pd1	0.0000	0.0000	0.0000	0.02259 (4)	
Cl1	0.22877 (4)	0.04262 (5)	0.15119 (3)	0.03461 (8)	
Cl2	0.07205 (5)	0.20056 (5)	−0.11116 (4)	0.03905 (9)	
O1	0.13229 (12)	−0.01272 (13)	0.62240 (10)	0.0298 (2)	
P1	0.21946 (4)	0.12971 (5)	0.59240 (3)	0.02481 (7)	
N1	0.15898 (16)	−0.05812 (19)	0.38798 (13)	0.0316 (3)	
H11	0.066 (3)	−0.046 (3)	0.384 (2)	0.056 (6)*	
H12	0.167 (2)	−0.064 (3)	0.317 (2)	0.055 (6)*	
H13	0.193 (2)	−0.151 (3)	0.421 (2)	0.049 (6)*	
C1	0.1204 (2)	0.3284 (2)	0.57050 (18)	0.0448 (4)	
H1A	0.0251	0.3174	0.5064	0.067*	
H1B	0.1807	0.4155	0.5515	0.067*	
H1C	0.1019	0.3595	0.6418	0.067*	
C2	0.40239 (19)	0.1665 (2)	0.69942 (15)	0.0422 (4)	
H2A	0.3919	0.2038	0.7725	0.063*	
H2B	0.4538	0.2533	0.6708	0.063*	
H2C	0.4604	0.0625	0.7128	0.063*	
C3	0.25318 (18)	0.0838 (2)	0.45344 (14)	0.0348 (3)	
H3A	0.2311	0.1854	0.4039	0.042*	
H3B	0.3596	0.0555	0.4713	0.042*	

### Atomic displacement parameters (Å^2^)

**Table:**

	*U*^11^	*U*^22^	*U*^33^	*U*^12^	*U*^13^	*U*^23^
Pd1	0.02378 (7)	0.02169 (7)	0.02289 (7)	−0.00035 (5)	0.00878 (5)	−0.00143 (5)
Cl1	0.02834 (16)	0.0438 (2)	0.02818 (16)	−0.00517 (14)	0.00520 (13)	−0.00045 (14)
Cl2	0.0447 (2)	0.0374 (2)	0.03567 (19)	−0.00905 (16)	0.01464 (16)	0.00673 (15)
O1	0.0301 (5)	0.0308 (5)	0.0315 (5)	−0.0009 (4)	0.0142 (4)	0.0028 (4)
P1	0.02492 (16)	0.02507 (16)	0.02430 (15)	0.00067 (12)	0.00827 (12)	0.00136 (12)
N1	0.0337 (7)	0.0324 (6)	0.0328 (6)	−0.0007 (5)	0.0170 (5)	−0.0036 (5)
C1	0.0529 (10)	0.0314 (8)	0.0572 (11)	0.0111 (7)	0.0280 (9)	0.0087 (7)
C2	0.0354 (8)	0.0466 (10)	0.0369 (8)	−0.0086 (7)	0.0027 (6)	0.0016 (7)
C3	0.0358 (8)	0.0404 (8)	0.0336 (7)	−0.0094 (6)	0.0190 (6)	−0.0037 (6)

### Geometric parameters (Å, º)

**Table:**

Pd1—Cl2^i^	2.3030 (4)	N1—H12	0.88 (2)
Pd1—Cl2	2.3030 (4)	N1—H13	0.84 (2)
Pd1—Cl1^i^	2.3049 (4)	C1—H1A	0.9600
Pd1—Cl1	2.3049 (4)	C1—H1B	0.9600
O1—P1	1.4951 (10)	C1—H1C	0.9600
P1—C2	1.7749 (16)	C2—H2A	0.9600
P1—C1	1.7809 (17)	C2—H2B	0.9600
P1—C3	1.8345 (15)	C2—H2C	0.9600
N1—C3	1.465 (2)	C3—H3A	0.9700
N1—H11	0.86 (2)	C3—H3B	0.9700
			
Cl2^i^—Pd1—Cl2	180.0	P1—C1—H1A	109.5
Cl2^i^—Pd1—Cl1^i^	88.803 (14)	P1—C1—H1B	109.5
Cl2—Pd1—Cl1^i^	91.196 (14)	H1A—C1—H1B	109.5
Cl2^i^—Pd1—Cl1	91.197 (14)	P1—C1—H1C	109.5
Cl2—Pd1—Cl1	88.803 (14)	H1A—C1—H1C	109.5
Cl1^i^—Pd1—Cl1	180.0	H1B—C1—H1C	109.5
O1—P1—C2	114.67 (7)	P1—C2—H2A	109.5
O1—P1—C1	112.61 (7)	P1—C2—H2B	109.5
C2—P1—C1	106.82 (9)	H2A—C2—H2B	109.5
O1—P1—C3	110.58 (7)	P1—C2—H2C	109.5
C2—P1—C3	105.30 (8)	H2A—C2—H2C	109.5
C1—P1—C3	106.27 (9)	H2B—C2—H2C	109.5
C3—N1—H11	111.1 (15)	N1—C3—P1	112.01 (10)
C3—N1—H12	108.8 (15)	N1—C3—H3A	109.2
H11—N1—H12	112 (2)	P1—C3—H3A	109.2
C3—N1—H13	110.2 (15)	N1—C3—H3B	109.2
H11—N1—H13	109 (2)	P1—C3—H3B	109.2
H12—N1—H13	106 (2)	H3A—C3—H3B	107.9
			
O1—P1—C3—N1	13.59 (14)	C1—P1—C3—N1	−108.92 (13)
C2—P1—C3—N1	137.98 (13)		

Symmetry code: (i) −*x*, −*y*, −*z*.

### Hydrogen-bond geometry (Å, º)

**Table:**

*D*—H···*A*	*D*—H	H···*A*	*D*···*A*	*D*—H···*A*
N1—H12···Cl1	0.88 (2)	2.40 (2)	3.2220 (15)	155 (2)
N1—H11···O1^ii^	0.86 (2)	1.89 (2)	2.7425 (17)	172 (2)
N1—H13···Cl1^iii^	0.84 (2)	2.73 (2)	3.3752 (15)	135.1 (18)
N1—H13···Cl2^iii^	0.84 (2)	2.82 (2)	3.5241 (16)	143.4 (18)

Symmetry codes: (ii) −*x*, −*y*, −*z*+1; (iii) −*x*+1/2, *y*−1/2, −*z*+1/2.
